# Intracoronary Imaging Implementation Gap in Contemporary PCI: The OCT2ACT Study

**DOI:** 10.1016/j.jacadv.2026.102982

**Published:** 2026-07-17

**Authors:** Simone Biscaglia, Daniela Trabattoni, Paolo Canova, Alberto Boi, Francesco Bruno, Giorgio Caretta, Flavio Biccirè, Alessio Mattesini, Gerlando Preti, Iacopo Muraca, Francesco Burzotta, Enrico Cerrato, Giandomenico Mancini, Sara Secchi, Giuseppe Tarantini, Mariangela Cicala, Gabriele Pesarini, Monica Verdoia, Jacopo Farina, Giuseppe Vadalà, Rocco Vergallo

**Affiliations:** a Azienda Ospedaliero Universitaria di Ferrara, Cona, Italy; b Centro Cardiologico Monzino, IRCCS, Milano, Italy; c ASST Papa Giovanni XXIII, Piazza OMS - Organizzazione Mondiale della Sanità, Bergamo, Italy; d Ospedale Arnas G. Brotzu, Cagliari, Italy; e A.O.U. Città della Salute e della Scienza di Torino-Ospedale Molinette, Torino, Italy; f Ospedale Civile Sant’Andrea, La Spezia, Italy; g Azienda Ospedaliera San Giovanni Addolorata, Roma, Italy; h Azienda Ospedaliero Universitaria Careggi -Interventistica Cardiologica Strutturale, Firenze, Italy; i Ospedale di Conegliano - ULSS 2 Marca Trevigiana, Conegliano, Italy; j Azienda Ospedaliero Universitaria Careggi- Cardiologia Interventistica d’urgenza, Firenze, Italy; k Policlinico A. Gemelli – Università Cattolica del Sacro Cuore, Roma, Italy; l Centro Unico di Emodinamica Rivoli-San Luigi Orbassano, Strada Rivalta, Rivoli-Orbassano, Italy; m Ospedale Generale Regionale “F. Miulli”, Acquaviva delle Fonti, Italy; n Policlinico Universitario Monserrato “Duilio Casula”, Monserrato, Italy; o Azienda Ospedale- Università Padova, Padova, Italy; p Ospedale “Di Venere” - Carbonara di Bari, Bari, Italy; q Azienda Ospedaliera Universitaria Integrata Verona, Verona, Italy; r Ospedale degli Infermi Via dei Ponderanesi, Ponderano, Italy; s Azienda Ospedaliero Universitaria Policlinico Paolo Giaccone, Palermo, Italy; t Ospedale Policlinico San Martino, Genova, Italy

**Keywords:** guidelines adherence, implementation science, intracoronary imaging, intravascular ultrasound, optical coherence tomography, percutaneous coronary intervention

## Abstract

**Background:**

Intracoronary imaging improves outcomes in complex percutaneous coronary intervention (PCI), yet its implementation in routine practice remains inconsistent.

**Objectives:**

This study sought to quantify the implementation gap for intracoronary imaging in contemporary PCI, identify operator-reported barriers to its use when predefined guideline-based anatomic criteria were present, and characterize modality selection beyond those criteria.

**Methods:**

OCT2ACT was a prospective, multicenter study enrolling 1,189 consecutive patients across 23 Italian imaging-capable centers between September 15 and December 15, 2025. Patients were classified as follows: cohort 1, predefined class IA anatomic criteria with no imaging; cohort 2, predefined class IA anatomic criteria with imaging; and cohort 3, imaging use outside these criteria.

**Results:**

Among 908 patients meeting predefined class IA criteria, 481 (53.0%) did not undergo intracoronary imaging. The most frequent barriers were operator-reported attitudinal factors, with angiography considered sufficient or imaging judged unlikely to change PCI strategy in 278 patients (57.8%). Imaging uptake varied substantially across centers (16.7% to 100.0%). In cohort 2, IVUS was the predominant modality (72%), whereas in cohort 3 optical coherence tomography was more common (54%), mainly for diagnostic clarification in ambiguous acute coronary syndromes or stent failure.

**Conclusions:**

Even in imaging-capable centers, intracoronary imaging remains underused in anatomically complex PCI. This implementation gap appears driven predominantly by operator attitudes and intersite practice variation rather than by cost or technical barriers alone. (Barriers Limiting OCT Penetration in Clinical Practice [OCT2ACT]; NCT07193693)

Percutaneous coronary intervention (PCI) has evolved significantly with the integration of intracoronary imaging, which enables more precise lesion characterization, optimization of stent implantation, confirmation that predefined procedural endpoints have been achieved, and recognition of stent-related complications. Contemporary 2024 European and 2025 American guidelines now support intravascular ultrasound (IVUS) and optical coherence tomography (OCT) for complex PCI with the highest level of recommendation in selected anatomical settings.^1^^,^^2^

Despite this strong endorsement and the growing evidence base supporting imaging-guided PCI,^1^, ^2^, ^3^, ^4^ real-world adoption remains inconsistent.^5^ While economic constraints and procedural time are often cited as barriers, no study has prospectively investigated the specific granular reasons for the underutilization of imaging following the release of the updated recommendations. Furthermore, there is a paucity of data regarding the use of intracoronary imaging, and particularly OCT, in anatomic subsets where it provides high diagnostic value but falls outside strict guideline mandates, such as in noncomplex bifurcations, specific patterns of severe calcification, or plaque characterization in acute coronary syndrome (ACS) patients.

Therefore, the aim of this study was to prospectively evaluate the penetration of intracoronary imaging in a real-world setting following the guideline upgrade. Specifically, we sought to: 1) quantify the current rate of imaging adoption in complex PCI; 2) identify the specific operator-reported reasons for not utilizing imaging when indicated; and 3) characterize the patterns of imaging use in clinical scenarios extending beyond current Class Ia recommendations.

## Methods

### Study organization

OCT2ACT (Assessment of Barriers Limiting Optical Coherence Tomography Penetration in Actual Clinical Practice & Training; NCT07193693) is an observational, prospective, multicenter, investigator-driven study that was conducted within a nationwide educational initiative promoting intracoronary imaging adoption, in particular OCT.^6^ Centers participating in the study were selected among Italian centers with availability of both imaging techniques (IVUS and OCT) and performing at least 20 OCT/year and 350 PCI/year. Initially, 45 centers were invited to participate in the study. Among those centers, 25 were willing to participate, but in 2 centers it was not possible to start the study because of administrative reasons. Therefore, 23 centers actively enrolled patients in the study. The present study is configured as a post-marketing observational study on device. For this reason, the UE 2017/745 law can be applied, and the approval of the Coordinating Center Ethics Committee was obtained before beginning the study. To this hand, the study protocol and the informed consent form used at the site and other appropriate documents were submitted and approved by the local Ethics Committee or Institutional Review Board and the appropriate regulatory authorities according to local legal requirements. The approval from the Coordinating Center Ethics Committee was applied to all the participating centers, according to the law. The sponsor of the study was the no profit entity Consorzio Futuro in Ricerca.^7^ The study was investigator driven with an unrestricted grant from Abbott Vascular (Santa Clara, USA). The funding source had no involvement in the design or conduct of the study; the collection, management, analysis, or interpretation of the data; the preparation, review, or approval of the manuscript; or the decision to submit the manuscript for publication. Full responsibility for the study design, data integrity, and reporting rests with the principal investigator and the steering committee.

### Study population

Participating centers prospectively enrolled consecutive eligible patients undergoing coronary angiography and/or PCI during a predefined 3-month study window in order to minimize patient-level selection bias. Patients were categorized into 3 predefined cohorts based on the presence of guideline-based anatomic indications for intracoronary imaging and the actual use of imaging during the index procedure.

#### Cohort 1 (Class Ia indication, imaging not used)

Patients undergoing PCI without intracoronary imaging despite the presence of at least one class IA anatomic criterion, defined as: long lesion (>38 mm), true bifurcation (reference vessel diameter >2.5 mm and Medina 1.1.1), or left main (LM) bifurcation.

#### Cohort 2 (Class Ia indication, imaging used)

Patients undergoing PCI with intracoronary imaging in the presence of at least one of the same predefined class IA anatomic criteria.

For the purpose of this study, Class Ia indications were operationalized using the 3 most objective and reproducible anatomic criteria explicitly referenced in contemporary European Society of Cardiology (ESC) guidelines (long lesion, true bifurcation, and LM bifurcation) in order to ensure standardized classification across centers and minimize interpretative variability.

The 3 predefined criteria were selected a priori because they were objective, angiographically reproducible, and directly aligned with the complex PCI subsets explicitly emphasized in the 2024 ESC guideline recommendation available when the protocol was finalized. This operational definition was intentionally conservative and was not intended to capture every contemporary scenario in which imaging may be recommended or clinically useful. Renal failure was defined in the electronic case report form as chronic kidney disease with estimated glomerular filtration rate <60 mL/min/1.73 m^2^ or chronic dialysis.

#### Cohort 3 (no Class Ia indication, imaging used)

Patients undergoing intracoronary imaging (with or without PCI) in the absence of the above predefined Class Ia anatomic criteria.

The only exclusion criterion was refusal to participate or inability to provide written informed consent.

### Data collection

All study data are anonymous and collected in a web-based electronical case report form.^8^ An academic research organization monitored integrity and quality of the data (We4 ClinicalResearch). Operators completed the study-specific fields immediately after the procedure in order to reduce recall bias. Imaging acquisition, interpretation, and procedural decision-making were left to local routine practice; no central imaging core laboratory was planned because the primary focus of the study was implementation behavior rather than image reinterpretation.

The educational initiative provided general training on intracoronary imaging, but the present registry did not prospectively collect center-level or operator-level imaging use before and after the training program. Therefore, OCT2ACT cannot quantify a pre-post educational effect or formally classify individual operators as persistent high- or low-imaging users. The free-text field was intended only to clarify the structured response when needed and was not designed as a qualitative substudy; consequently, free-text comments are summarized descriptively and not analyzed as a formal thematic data set.

### Primary endpoints

The primary objectives of the study were to investigate: 1) the main barriers limiting the use of intracoronary imaging in cohort 1; 2) the main reasons behind the selection of IVUS or OCT in cohort 2 and 3; 3) safety of imaging (in particular OCT) utilization in cohort 3. Barriers to imaging use were prospectively captured using a structured, predefined questionnaire completed by the primary operator immediately after the procedure. Consistent with the conceptual framework proposed by Cabana et al,^9^ reported reasons were classified into 3 prespecified domains: 1) attitudinal barriers (lack of agreement with the recommendation or inertia of previous practice); 2) knowledge-related barriers (lack of awareness or familiarity); and 3) behavioral or external barriers (including logistical, economic, or time constraints) (Table 1). Operators were required to identify the primary reason for imaging avoidance, with the option to indicate additional contributing factors. Responses were centrally adjudicated and categorized according to the predefined framework to ensure uniform classification across centers.

**Table 1:** Classification of Barriers Leading to Imaging Avoidance

Attitudes (Lack of Agreement and Inertia of Previous Practice)	Knowledge (Lack of Awareness and Familiarity)	Behavior (External Barriers)
Clinical and angiographic data are sufficient	Intracoronary imaging not feasible for technical or anatomical reasons (eg, tortuous vessels, etc)	Time constraint
Intracoronary imaging would not change my PCI strategy	Lack of confidence in interpretation of images	Device costs
Intracoronary imaging has low added value on improving clinical outcome	Lack of confidence in translating imaging features into an actionable PCI plan	Absence of adequate coding/reimbursement
	Inadequate support by device specialists in the cath lab	

PCI = percutaneous coronary intervention.

### Secondary endpoints

Major secondary endpoints were any correlations between angiographic features and imaging disuse. Other secondary endpoints included: 1) penetration of intracoronary imaging among procedures fulfilling predefined Class Ia anatomic criteria; 2) the percentage of imaging-guided procedures relative to overall PCI volume and to predefined class IA-eligible PCI volume; 3) associations between baseline and procedural characteristics and imaging nonuse among class IA-eligible cases; and 4) associations between baseline and procedural characteristics and modality selection (IVUS vs OCT) in imaging-guided procedures. Exploratory post hoc analyses also assessed site-level heterogeneity in imaging uptake among class IA-eligible cases, weekday vs weekend imaging use, and procedural outcomes among patients who actually underwent PCI. The study protocol is reported in the Supplemental Appendix.

### Safety endpoints

Safety endpoints were assessed in imaging-guided procedures and included total procedural time, fluoroscopy time, contrast volume, imaging failure, and predefined intraprocedural or periprocedural complications potentially related to imaging use. Safety data were recorded in both imaging cohorts and compared descriptively. All adverse events were systematically captured in the electronic case report form and independently reviewed to determine their relationship to imaging.

### Sample size and statistical analysis

Given the observational and exploratory nature of the study, no formal hypothesis-driven sample size calculation was performed. The study was instead designed to provide a precision-based estimate of intracoronary imaging penetration in contemporary practice following the guideline upgrade. Based on feasibility considerations, participating centers were expected to include at least 12 high-volume hubs performing ≥70 PCI procedures per month. According to predefined eligibility criteria, each center was anticipated to contribute approximately 30 complex PCI cases per month over the 3-month consecutive enrollment window, yielding an expected sample size of at least 1,000 patients. This sample size was deemed sufficient to provide stable estimates of imaging utilization rates and to allow exploratory multivariable modeling of factors associated with imaging avoidance. Continuous variables were summarized as mean (SD) or median [IQR], according to distribution assessed with the Shapiro-Wilk test. Categorical variables were presented as frequencies and percentages and compared using Pearson chi-square or Fisher exact tests, as appropriate. Multivariable logistic regression models were prespecified as exploratory models. Candidate variables were selected a priori on the basis of clinical plausibility and availability in the electronic case report form, including patient age, operator age, major comorbidities, renal failure, and treated vessel location; no automated stepwise model-building procedure was used. Age was modeled per 10-year increment for interpretability. Unadjusted associations for variables included in the multivariable model are reported in the Supplemental Appendix. Site-level heterogeneity was summarized descriptively by center-level imaging uptake among class IA-eligible cases. Statistical analyses were performed using STATA version 16 and Python. Two-tailed analyses were performed, and a *P* value <0.05 was considered significant.

## Results

**Figure 1:**
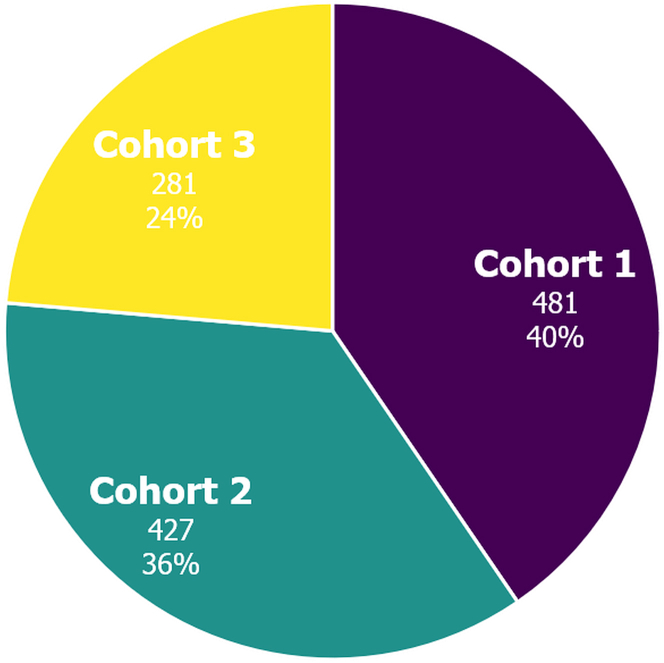
Study Cohorts

From September 15 to December 15, 2025, a total of 1,189 consecutive patients were enrolled across 23 centers (Figure 1). Overall, 481 patients (40%) were included in cohort 1 (Class Ia indication, imaging not used), 427 (36%) in cohort 2 (Class Ia indication, imaging used), and 281 (24%) in cohort 3 (no Class Ia indication, imaging used). Among the 908 patients fulfilling Class Ia anatomic criteria, 481 (53.0%) did not undergo intracoronary imaging, whereas 281 of 708 total imaging procedures (39.7%) were performed outside class IA indications. Baseline and procedural characteristics across the 3 cohorts are summarized in Tables 2 and 3. Imaging uptake among Class Ia-eligible cases varied markedly across centers, ranging from 16.7% to 100.0%, with a median center-level imaging rate of 49.2% (IQR: 37.8%-72.8%). In an exploratory calendar-based analysis, imaging penetration among class IA-eligible cases did not differ significantly between weekdays and weekends (47.4% vs 41.9%; *P* = 0.484).

**Table 2:** Baseline Patient Characteristics

	Total (N = 1,189)	Cohort 1 (n = 481)	Cohort 2 (n = 427)	Cohort 3 (n = 281)	*P* Value
Patient age, y	69.5 (11.2)	70.5 (11.7)	70.1 (10.3)	67.0 (11.2)	<0.001
Patient male sex	979 (82.5%)	398 (82.9%)	357 (83.8%)	224 (80.0%)	0.41
Operator age, y	44.3 (9.6)	45.6 (9.6)	43.6 (9.5)	43.0 (9.4)	<0.001
Operator male sex	1,077 (90.8%)	437 (91.0%)	386 (90.6%)	254 (90.7%)	0.97
Hypertension	909 (76.6%)	377 (78.5%)	338 (79.3%)	194 (69.3%)	0.004
Dyslipidemia	865 (72.9%)	353 (73.5%)	324 (76.1%)	188 (67.1%)	0.031
Diabetes	285 (24.0%)	126 (26.3%)	105 (24.6%)	54 (19.3%)	0.089
Current smoker	205 (17.3%)	83 (17.3%)	68 (16.0%)	54 (19.3%)	0.52
Former smoker	352 (29.7%)	144 (30.0%)	124 (29.1%)	84 (30.0%)	0.95
Previous MI	284 (23.9%)	100 (20.8%)	104 (24.4%)	80 (28.6%)	0.052
Previous PCI	379 (32.0%)	126 (26.3%)	149 (35.0%)	104 (37.1%)	0.002
CABG	47 (4.0%)	30 (6.3%)	13 (3.1%)	4 (1.4%)	0.002
AF	104 (8.8%)	44 (9.2%)	37 (8.7%)	23 (8.2%)	0.90
COPD	81 (6.8%)	34 (7.1%)	27 (6.3%)	20 (7.1%)	0.88
PAD	137 (11.6%)	59 (12.3%)	57 (13.4%)	21 (7.5%)	0.046
Renal failure	182 (15.3%)	96 (20.0%)	66 (15.5%)	20 (7.1%)	<0.001
CVA	50 (4.2%)	27 (5.6%)	17 (4.0%)	6 (2.1%)	0.067
Clinical presentation					0.006
CCS	499 (42.1%)	189 (39.4%)	197 (46.2%)	113 (40.4%)	
NSTEMI	268 (22.6%)	109 (22.7%)	98 (23.0%)	61 (21.8%)	
STEMI	178 (15.0%)	96 (20.0%)	43 (10.1%)	39 (13.9%)	
UA	154 (13.0%)	55 (11.5%)	55 (12.9%)	44 (15.7%)	
Other	87 (7.3%)	31 (6.5%)	33 (7.7%)	23 (8.2%)	

Continuous variables are expressed as mean (SD).

AF = atrial fibrillation; CABG = coronary artery bypass graft; CCS = chronic coronary syndrome; COPD = chronic obstructive pulmonary disease; CVA = cerebrovascular accident; MI = myocardial infarction; NSTEMI = non-ST-segment elevation myocardial infarction; PAD = peripheral artery disease; PCI = percutaneous coronary intervention; STEMI = ST-segment elevation myocardial infarction; UA = unstable angina.

**Table 3:** Procedural Characteristics

	Total (N = 1,189)	Cohort 1 (n = 481)	Cohort 2 (n = 427)	Cohort 3 (n = 281)	*P* Value
Procedure duration, min	78.1 (40.9)	75.3 (36.2)	90.8 (41.5)	63.7 (42.0)	<0.001
X-ray time, min	24.4 (17.3)	24.4 (15.8)	28.2 (17.3)	18.6 (18.2)	<0.001
Contrast amount, mL	193.1 (113.6)	192.5 (135.6)	209.4 (99.6)	169.1 (83.9)	<0.001
Access site					0.41
Radial	1,033 (88.2%)	423 (88.7%)	363 (86.6%)	247 (89.8%)	
Femoral	138 (11.8%)	54 (11.3%)	56 (13.4%)	28 (10.2%)	
Complex criteria					
Long lesion	562 (48.0%)	348 (73.0%)	212 (50.6%)	2 (0.7%)	<0.001
True bifurcation	182 (15.5%)	83 (17.4%)	99 (23.6%)	0 (0.0%)	<0.001
LM bifurcation	323 (27.6%)	112 (23.5%)	209 (49.9%)	2 (0.7%)	<0.001
Invasive physiology use	41 (3.5%)	17 (3.6%)	15 (3.6%)	9 (3.3%)	0.97
PCI					
LM	341 (29.1%)	117 (24.5%)	211 (50.4%)	13 (4.7%)	<0.001
LAD	788 (67.3%)	308 (64.6%)	325 (77.6%)	155 (56.4%)	<0.001
LCx	309 (26.4%)	139 (29.1%)	123 (29.4%)	47 (17.1%)	<0.001
RCA	252 (21.5%)	134 (28.1%)	64 (15.3%)	54 (19.6%)	<0.001
Ramus	30 (2.6%)	11 (2.3%)	15 (3.6%)	4 (1.5%)	0.20
Graft	3 (0.3%)	2 (0.4%)	1 (0.2%)	0 (0.0%)	0.55
None	64 (5.5%)	3 (0.6%)	12 (2.9%)	49 (17.8%)	<0.001
Successful PCI	1,116 (95.3%)	470 (98.5%)	414 (98.8%)	232 (84.4%)	<0.001

Continuous variables are expressed as mean (SD).

LAD = left anterior descending; LCx = left circumflex; LM = left main; N = number; RCA = right coronary artery; other abbreviation as in Table 2.

Patients in cohort 1 were older. Patients in cohort 2 more frequently exhibited a higher-risk clinical and anatomic profile, with a greater burden of cardiovascular risk factors and prior coronary disease, a higher proportion of STEMI presentations, and more frequent LM PCI if compared with cohort 1. Cohort 3 included patients with lower anatomic complexity by the predefined Class Ia criteria (Tables 2 and 3).

### Cohort 1 (class IA indication, imaging not used)

**Figure 2:**
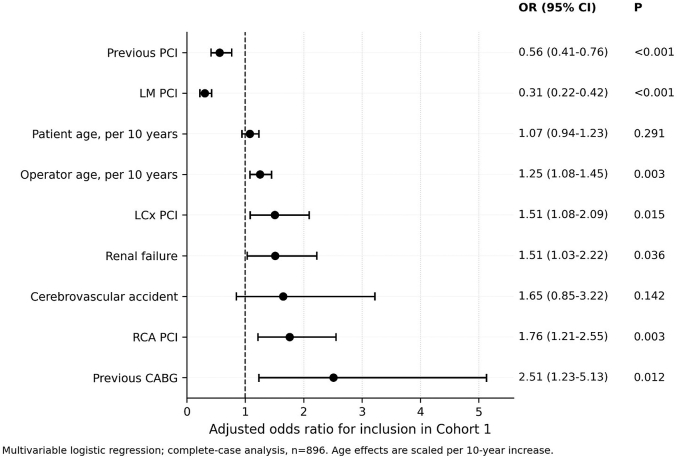
Predictors of Imaging Nonuse ORs are shown for likelihood of inclusion in cohort 1 among Class Ia-eligible cases. Age variables are expressed per 10-year increment and displayed on a regular OR scale. CABG = coronary artery bypass graft; LCx = left circumflex; LM = left main; PCI = percutaneous coronary intervention; RCA = right coronary artery.

At multivariable analysis, older operator age, previous coronary artery bypass grafting, renal failure, PCI on left circumflex artery, and absence of LM PCI were independently associated with inclusion in cohort 1 (Figure 2). Unadjusted associations for all variables entered in the multivariable model are provided in Supplemental Table 4.

**Figure 3:**
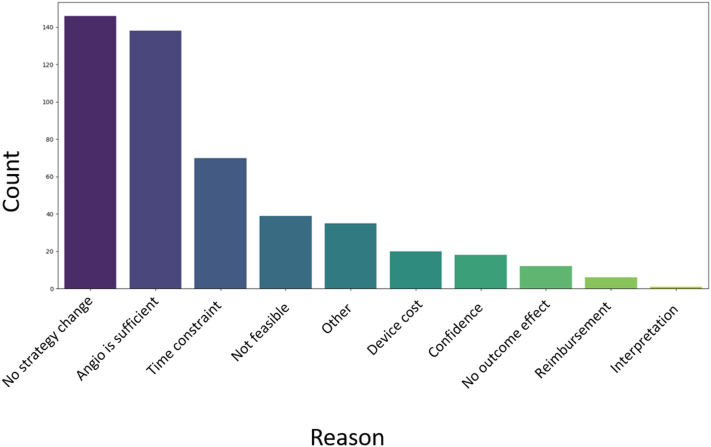
Main Barrier for Imaging Avoidance in Cohort 1

Reasons for not performing imaging in cohort 1 are reported in Supplemental Figure 1. When asked about the main reason, the most frequent reported barriers were attitudinal: sufficiency of angiographic information to guide treatment in 138 patients (28.7%) and the expectation that imaging would not change PCI strategy in 140 patients (29.1%). Taken together, these 2 responses accounted for 278 of 481 cases (57.8%). Behavioral or external barriers accounted for a minority of cases with time constraints or device costs reported in 90 patients (18.7%) (Figure 3).

### Cohort 2 (class IA indication, imaging used)

In cohort 2, imaging use was primarily driven by adherence to guideline recommendations (61%), operator experience (67%), and the conviction that imaging would improve procedural outcomes in the individual patient (78%). At multivariable analysis, PCI involving left anterior descending or LM artery independently predicted inclusion in cohort 2, whereas increasing operator age was negatively associated with imaging use (Supplemental Figure 2). Within this cohort, IVUS was the predominant modality used (308 patients, 72%), while OCT was used in 121 patients (28%). IVUS was preferentially selected in ostial lesions (45%) and in patients with chronic kidney disease (14%), whereas OCT was more frequently chosen in bifurcation lesions (50%), severe calcification (40%), and stent failure (12%). Imaging-guided procedures were associated with greater procedural burden compared with cohort 1, including longer procedural duration and higher contrast utilization (*P* < 0.001 and *P* = 0.037, respectively). Conversely, procedural time and contrast use were lower in cohort 3 compared with cohort 1 (both *P* < 0.001). Imaging could not be completed in 6 patients (3 uncrossable lesions, 2 probe damage, and 1 device entrapment). Intraprocedural or periprocedural complications occurred in 14 patients (3.2%), with detailed reporting provided in Supplemental Table 1.

### Cohort 3 (no class IA indication, imaging used)

The main reason for inclusion in cohort 3 was to improve diagnostic clarification (188 patients, 67%). At multivariable analysis, operator age, renal failure, and PCI on the LM were inversely associated with inclusion in cohort 3, whereas previous PCI was positively associated (Supplemental Figure 3). In cohort 3, OCT was selected as an imaging technique more frequently than IVUS (n = 152, 54% vs n = 129, 46%, respectively). The main clinical reasons for OCT utilization were stent failure (n = 39, 26%) or ambiguous ACS (n = 52, 34%), whereas the main technical reason was the perceived need for higher spatial resolution (n = 82, 54%).

### Safety endpoint

In cohort 3, procedural or periprocedural complications occurred in 10 patients (3.5%), including 6 cases (3.9%) in the OCT group and 4 cases (3.1%) in the IVUS group. Rates did not differ significantly compared with cohort 2 (*P* = 0.84) or between OCT and IVUS (*P* = 0.76). None of the recorded events were adjudicated as directly attributable to the imaging device itself. Detailed event descriptions are provided in Supplemental Table 2. In 2 cases, OCT acquisition was incomplete: one due to inability to cross a previously implanted stent and one due to device malfunction.

## Discussion

The main results of the OCT2ACT study are: 1) more than one-half of patients fulfilling predefined class IA anatomic criteria underwent PCI without intracoronary imaging; 2) the predominant barriers to imaging adoption were operator-reported attitudinal factors rather than purely external constraints such as reimbursement or technical limitations; 3) imaging uptake showed marked heterogeneity across operators and centers, with a tendency for lower imaging utilization for older operators; 4) when imaging was used outside predefined Class Ia criteria, OCT was preferentially selected for diagnostic clarification, particularly in stent failure and ambiguous ACS presentations (Central Illustration).

**Central Illustration:**
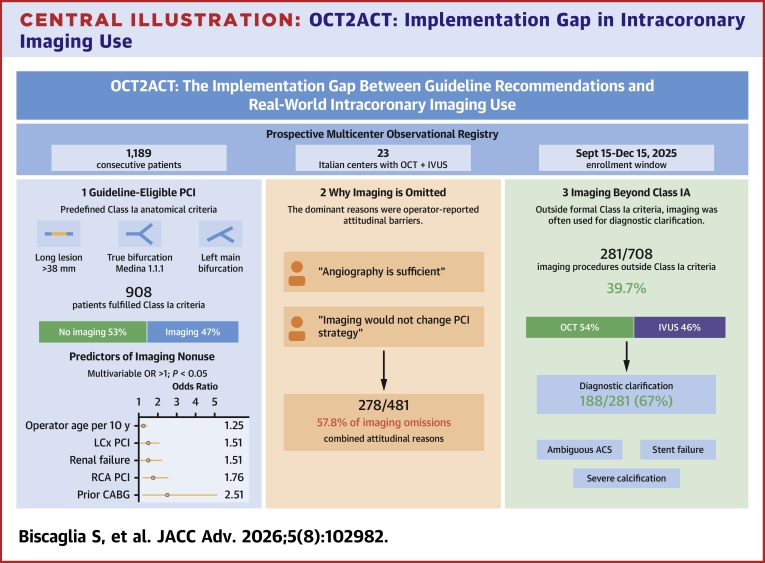
OCT2ACT: Implementation Gap in Intracoronary Imaging Use In this prospective, multicenter observational registry, 1,189 consecutive patients were enrolled across 23 Italian centers with availability of both optical coherence tomography (OCT) and intravascular ultrasound (IVUS). Among 908 patients fulfilling predefined Class Ia anatomic criteria for intracoronary imaging, 481 (53%) underwent PCI without imaging and 427 (47%) received imaging-guided PCI. Imaging nonuse was mainly associated with operator-reported attitudinal barriers, particularly the perception that angiography was sufficient or that imaging would not modify PCI strategy, accounting for 278 of 481 missed imaging opportunities (57.8%). Predictors of imaging nonuse included older operator age, prior CABG, renal failure, LCx PCI, and RCA PCI. Among imaging procedures performed outside predefined Class Ia criteria, OCT was used more frequently than IVUS and was mainly selected for diagnostic clarification in ambiguous ACS, stent failure, and severe calcification. ACS = acute coronary syndrome; IVUS = intravascular ultrasound; OCT = optical coherence tomography; other abbreviations as in Figure 1.

OCT2ACT identifies a substantial implementation gap between class IA guideline recommendations and contemporary PCI practice. Despite strong endorsement from both European and American guidelines,^10^^,^^11^ more than half of patients meeting predefined Class Ia anatomic criteria did not undergo intracoronary imaging. Importantly, this pattern was observed in imaging-capable centers, suggesting that structural availability alone is insufficient to ensure implementation of evidence-based strategies.

These findings align with prior registry data indicating that the transition from angiography-guided to imaging-guided PCI remains incomplete, even in the presence of robust randomized evidence supporting improved outcomes and cost-effectiveness.^5^^,^^12^, ^13^, ^14^ Together, these data underscore that guideline endorsement does not automatically translate into routine clinical adoption, highlighting the complexity of implementation in interventional cardiology.^15^^,^^16^ The recent results from OPTIMAL (Optimization of Left Main PCI With Intravascular Ultrasound) and IVUS-CHIP (Intravascular Ultrasound Guidance for Complex High-risk Indicated Procedures) trials suggest that the benefit of imaging is proportional to the extent to which imaging findings translate into meaningful procedural modification rather than image acquisition alone.^17^^,^^18^

A central insight of OCT2ACT is that imaging underuse appears to be predominantly driven by operator-level behavioral determinants rather than by economic or logistical constraints. The most frequently reported reasons for imaging avoidance were related to perceived sufficiency of angiographic assessment and the expectation that imaging would not meaningfully alter procedural strategy. This suggests a persistent reliance on visual angiographic estimation and established practice patterns, even in anatomically complex scenarios where intravascular guidance is recommended. Interestingly, when the same level of indication (Class Ia) was provided for intracoronary physiology, a prospective registry demonstrated a similar attitude of the operators toward physiology.^19^

Operator age emerged as an independent factor of imaging avoidance. While this association should not be interpreted as causal, it may reflect differences in training background and exposure to imaging-guided paradigms during formative years of practice. Notably, previous large-scale analyses from the United States have demonstrated substantial interhospital variability in imaging use that was not fully explained by patient or procedural characteristics.^20^

Beyond operator-related determinants, certain patient characteristics, including prior coronary artery bypass grafting, cerebrovascular disease, and renal dysfunction, were associated with imaging avoidance. These findings may reflect a certain degree of inertia in the use of imaging in patients perceived as frail or clinically high risk. However, contemporary data indicate that complex and high-risk subsets may derive at least comparable, if not greater, benefit from evidence-based revascularization strategies.^21^, ^22^, ^23^ This highlights the need to better align perceived procedural burden with objective risk–benefit considerations. An additional relevant observation is that 40% of imaging procedures were performed outside formal Class Ia indications. In these cases, OCT was the preferred imaging modality, particularly in scenarios requiring diagnostic clarification and procedural guidance such as stent failure, severe calcifications, or ambiguous ACS presentations. This pattern suggests that clinical practice may be evolving beyond the current scope of guideline-defined complexity, with operators leveraging high-resolution imaging for mechanistic insight rather than solely for stent optimization. Rather than representing inappropriate overuse, this finding may indicate areas where guideline recommendations have not yet fully incorporated emerging diagnostic applications. Procedural burden was higher in imaging-guided class IA cases, as reflected by longer procedural duration and greater contrast use, consistent with prior literature.^24^ Nevertheless, imaging-related complications were infrequent and comparable across cohorts, supporting the procedural safety of both IVUS and OCT in contemporary practice.

Taken together, these findings suggest that improving penetration of imaging-guided PCI will probably require more than wider access to devices. Targeted operator education, standardized imaging training, prospective audit-feedback programs, and center-level benchmarking may all be necessary to close the evidence-to-practice gap. Future studies should focus not only on broader adoption but also on appropriate case selection, standardized optimization algorithms, and the extent to which imaging changes procedural decision-making in a way that ultimately improves clinical outcomes.

### Study limitations

This study has several limitations. First, it was conducted in centers with on-site availability of both IVUS and OCT and predefined minimum activity thresholds; therefore, generalizability to lower-resource or truly low-volume settings remains uncertain. Second, the study was embedded within an educational initiative focused on intracoronary imaging, and operators were aware that imaging use was being prospectively recorded; accordingly, a Hawthorne effect leading to higher imaging use than in unobserved routine practice cannot be excluded. Third, no preintervention or postintervention imaging-use data were collected, and no unique validated operator identifier was available for phenotype analyses; therefore, the study cannot quantify the effect of training or distinguish persistent operator-level behavior from center-level practice patterns. Fourth, barriers were self-reported by operators and are therefore susceptible to reporting, justification, and social-desirability bias. Fifth, the predefined class IA criteria were deliberately conservative and did not include all contemporary scenarios in which imaging may be recommended or useful, including nonbifurcation LM PCI and severe calcification. Finally, comparisons of procedural success and complications across cohorts and between IVUS and OCT were exploratory, nonrandomized, and should not be interpreted causally.

## Conclusions

In contemporary PCI practice, a significant gap persists between Class Ia guideline recommendations for intracoronary imaging use to guide complex PCI and their actual implementation. This gap appears to be driven predominantly by operator-level behavioral factors rather than by structural constraints. At the same time, OCT is frequently employed beyond formal guideline indications for diagnostic refinement in complex clinical scenarios. Bridging the evidence-to-practice gap will likely require targeted educational, behavioral, and system-level interventions aimed at facilitating the consistent adoption of imaging-guided PCI.

Perspectives **COMPETENCY IN PATIENT CARE AND PROCEDURAL SKILLS:** In contemporary PCI practice, guideline-supported intracoronary imaging is still omitted in approximately one-half of anatomically eligible cases, most often because operators judge angiography sufficient or believe imaging is unlikely to modify procedural strategy.

**TRANSLATIONAL OUTLOOK:** Future prospective studies should determine whether standardized intracoronary imaging training, audit-feedback programs, and center-level benchmarking can reduce the implementation gap and whether broader uptake translates into measurable changes in PCI strategy and clinical outcomes.

## Funding support and author disclosures

The study received an unconditional grant from Abbott Vascular. This company had no involvement in the trial design, data collection, analysis, interpretation, or writing of the manuscript. Dr Biscaglia has received speaker fees from SMT, Medis, Siemens, and Abbott Vascular; has received institutional grants from SMT, Medis, Siemens, and Abbott Vascular; and has served as a member of the advisory board for Siemens and Abbott. Dr Canova is a consultant for Abbott Vascular and Boston Scientific; and has received grants from Amarin Pharma Inc, Amgen, Bristol Myers Squibb Company, Daiichi Sankyo, Sanofi, and Terumo. Dr Boi has received speaker fee from Abbott Vascular. Dr Flavio Biccirè has received speaking or consulting fees from Abbott, Sanofi, and Ultragenyx. Dr Tarantini has received lecture fees from Edwards Lifesciences, Abbott, Medtronic, SMT, and Johnson & Johnson. Dr Mattesini has received speaker fee from Abbott Vascular. Dr Francesco Burzotta has received speaker fees from Abbott, Abiomed, Edwards, Medtronic, and Terumo. Dr Francesco Bruno has received speaking honoraria from Abbott and Boston Scientific. Dr Cerrato has received an institutional research grant and speaker fees from Abbott. Dr Vergallo is a consultant for Abbott Vascular, Boston Scientific, and Medtronic; has received lecturing fees from Abbott Vascular, Abiomed, Amgen, Boston Scientific, Daiichi Sankyo, Edwards Lifesciences, Johnson & Johnson, Medtronic, Novartis, Novo Nordisk, Penumbra Inc., Philips, and SIS Medical; and has served as a member of advisory boards for Abbott Vascular, Amarin, Amgen, and Medtronic. All other authors have reported that they have no relationships relevant to the contents of this paper to disclose.
