# Supplementary material for: Intracoronary Imaging Implementation Gap in Contemporary PCI: The OCT2ACT Study
Source: JACC Adv. 2026 Jul 17;5(8):102982. doi: 10.1016/j.jacadv.2026.102982 (PMC13400869; doi:10.1016/j.jacadv.2026.102982)
Supplement: Supplemental Material [file mmc1.pdf]

SUPPLEMENTARY MATERIALS

Supplementary Table 1. Procedural and periprocedural complications in Cohort 2…………..2

Supplementary Table 2. Procedural and periprocedural complications in Cohort 3…………..3

Supplementary Table 3. Cardiovascular Therapy……………………………………………...4

Supplementary Figure 1. Reasons for not performing imaging (Cohort 1)……………………..5

Supplementary Figure 2. Odds ratio for likelihood of inclusion in Cohort 2………………..…6

Supplementary Figure 3. Odds ratio for likelihood of inclusion in Cohort 3…………………...7

Supplementary Table 1. Procedural and periprocedural complications in Cohort 2.

	**Cohort 2 (n=427)**
Complications	14 (3.3)
Perforation	4 (0.9)
Vessel occlusion/dissection	5 (1.2)
No reflow	1 (0.2)
Acute kidney injury	1 (0.2)
Acute heart failure	1 (0.2)
Cardiogenic shock	1 (0.2)
Femoral hematoma	1 (0.2)

Supplementary Table 2. Procedural and periprocedural complications in Cohort 3.

	**IVUS (n=129)**	**OCT (n=152)**	**P**
Complications	4 (3.1)	6 (3.9)	0.76
Acute kidney injury	1 (0.8)	0 (0)	
Femoral pseudo-aneurysm	1 (0.8)	0 (0)	
Ostial stent crush	1 (0.8)	0 (0)	
Side branch dissection/occlusion	0 (0)	2 (1.3)	
Stroke	0 (0)	1 (0.7)	
Periprocedural MI	0 (0)	1 (0.7)	
Microcatheter fracture	0 (0)	1 (0.7)	

IVUS: intravascular ultrasound. OCT: optical coherence tomography. MI: myocardial infarction.

Supplementary Table 3. Cardiovascular Therapy.

	Total N=1,189	Cohort 1 N=481	Cohort 2 N=427	Cohort 3 N=281	p-value
Aspirin	1,143 (97.6%)	470 (98.5%)	412 (98.3%)	261 (94.9%)	0.004
Clopidogrel	613 (52.3%)	259 (54.3%)	235 (56.1%)	119 (43.3%)	0.002
Ticagrelor	428 (36.5%)	181 (37.9%)	150 (35.8%)	97 (35.3%)	0.71
Prasugrel	73 (6.2%)	26 (5.5%)	23 (5.5%)	24 (8.7%)	0.15
Warfarin	13 (1.1%)	4 (0.8%)	6 (1.4%)	3 (1.1%)	0.70
NOAC	109 (9.3%)	42 (8.8%)	40 (9.5%)	27 (9.8%)	0.88
ACEi-ARB	733 (62.6%)	318 (66.7%)	259 (61.8%)	156 (56.7%)	0.023
B-blocker	697 (59.5%)	290 (60.8%)	260 (62.1%)	147 (53.5%)	0.060
Statin	1,034 (88.3%)	431 (90.4%)	362 (86.4%)	241 (87.6%)	0.17
Ezetimibe	910 (77.7%)	374 (78.4%)	331 (79.0%)	205 (74.5%)	0.35
Bempedoic acid	47 (4.0%)	16 (3.4%)	21 (5.0%)	10 (3.6%)	0.42
PCSK9	47 (4.0%)	13 (2.7%)	22 (5.3%)	12 (4.4%)	0.15

NOAC: novel oral anticoagulant. ACEi-ARB: angiotensin-converting enzyme inhibitors-Angiotensin II Receptor Blocker. PCSK9: Proprotein Convertase Subtilisin/Kexin type 9.

Supplementary figure 1. Reasons for not performing imaging (Cohort 1)

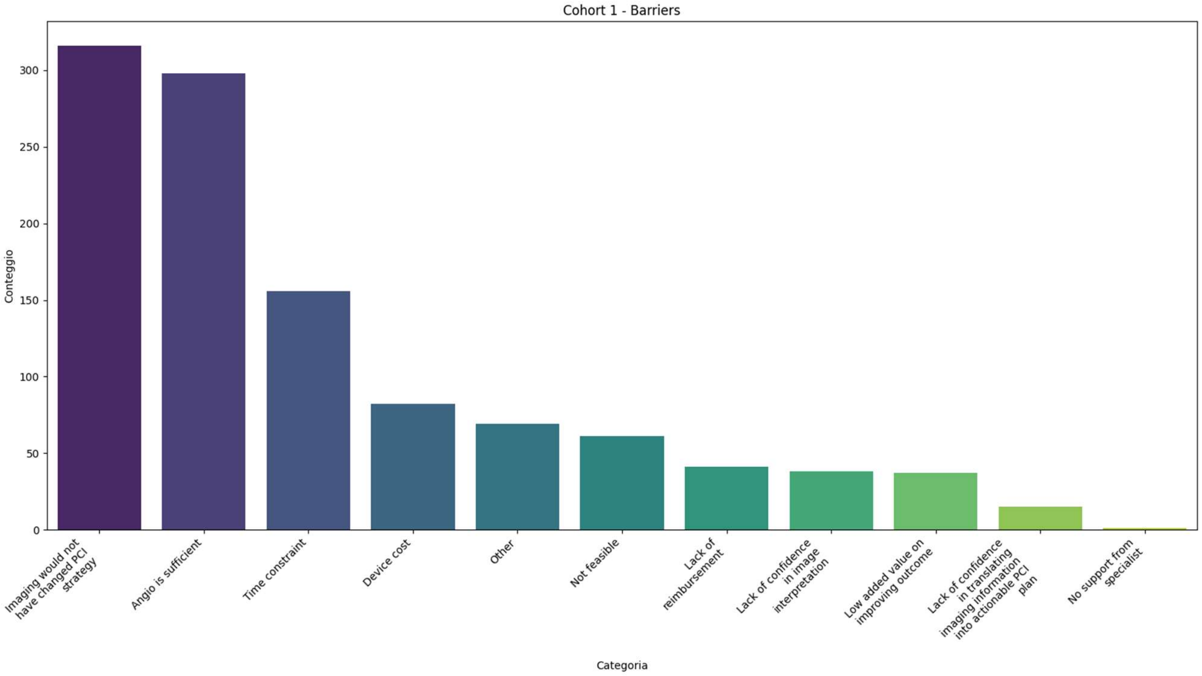

Supplementary Figure 2. Odds ratio for likelihood of inclusion in Cohort 2.

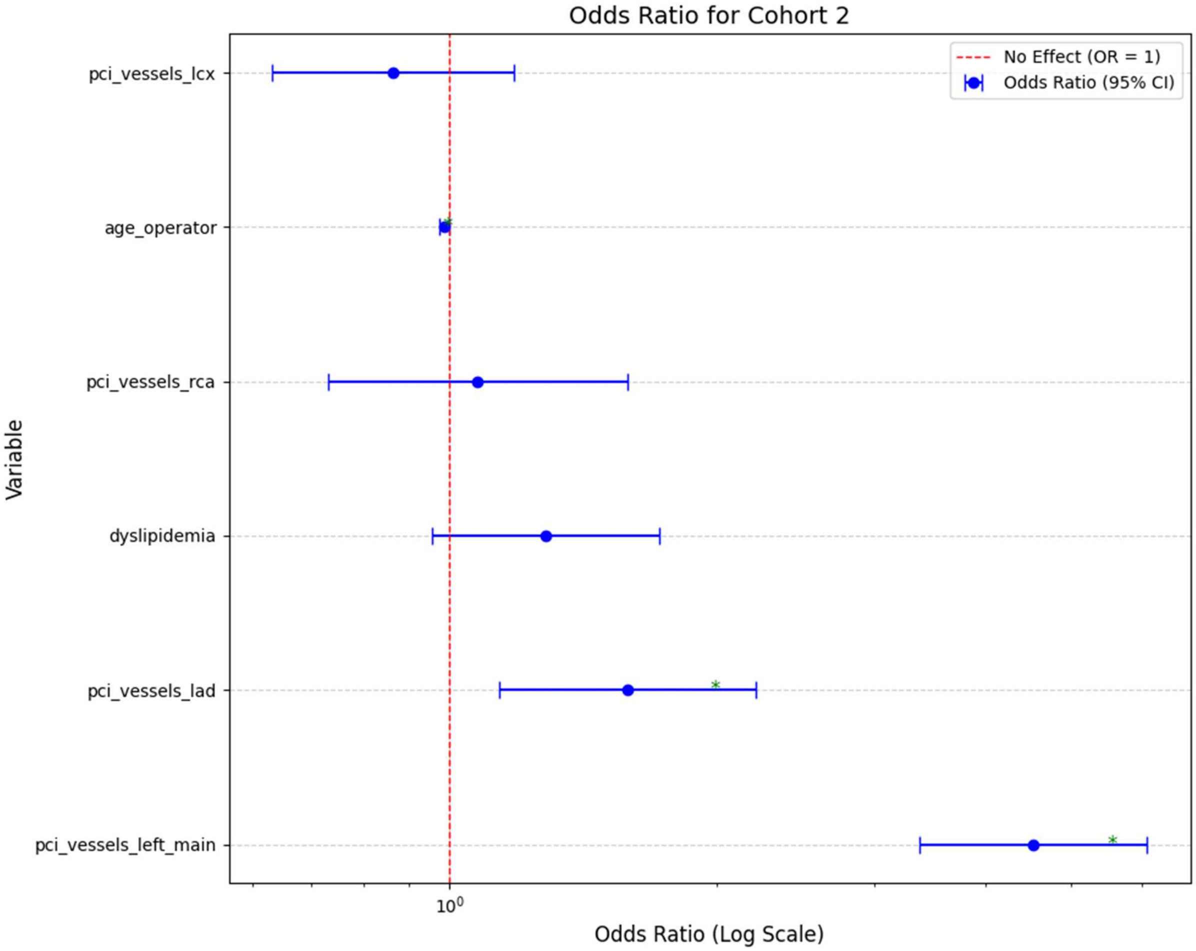

Supplementary Figure 3. Odds ratio for likelihood of inclusion in Cohort 3.

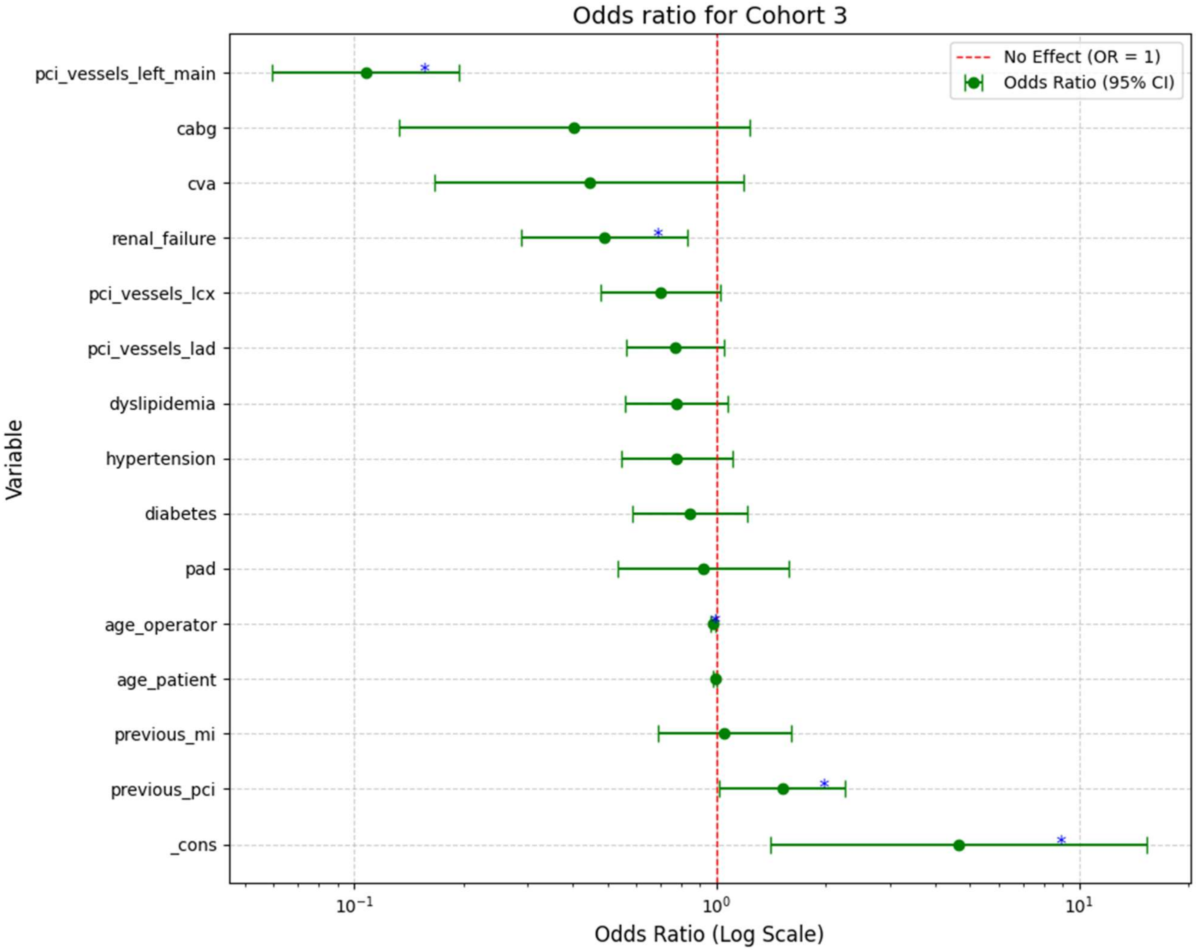

Supplementary Table 4. Unadjusted associations with imaging nonuse among Class IA-eligible cases.

Variable	No. analyzed	Unadjusted OR (95% CI)	P value
Patient age, per 10 years	906	1.04 (0.92-1.17)	0.526
Operator age, per 10 years	906	1.25 (1.09-1.44)	0.001
Prior CABG	906	2.12 (1.09-4.12)	0.027
CVA	906	1.43 (0.77-2.67)	0.256
Renal failure	906	1.36 (0.97-1.93)	0.078
RCA PCI	896	2.17 (1.55-3.02)	<0.001
LCx PCI	896	0.99 (0.74-1.32)	0.944
LAD PCI	896	0.53 (0.39-0.71)	<0.001
Left main PCI	896	0.32 (0.24-0.43)	<0.001

Odds ratios are for likelihood of inclusion in Cohort 1 versus Cohort 2. Age variables are expressed per 10-year increment. CABG: coronary artery bypass grafting; CVA: cerebrovascular accident; LAD: left anterior descending artery; LCx: left circumflex artery; OR: odds ratio; PCI: percutaneous coronary intervention; RCA: right coronary artery.

Supplementary Table 5. Center-level imaging uptake among Class IA-eligible cases.

Center	Class IA eligible cases	Imaged cases	Imaging uptake, %
Azienda Ospedaliera Santa Croce e Carle	114	19	16.7
Ospedale Generale Regionale "F. Miulli"	38	8	21.1
Policlinico Universitario Monserrato "Duilio Casula"	33	8	24.2
IRCCS Policlinico San Donato	9	3	33.3
Policlinico A. Gemelli	32	11	34.4
Ospedale Policlinico San Martino	90	32	35.6
Centro Cardiologico Monzino	105	42	40.0
P.O.S. Giuseppe Moscati Aversa	7	3	42.9
Ospedale Molinette Torino	67	29	43.3
Azienda Ospedaliera Universitaria Integrata Verona	17	8	47.1
Azienda Ospedaliera San Giovanni Addolorata	39	19	48.7
ASST Papa Giovanni XXIII	59	29	49.2
Azienda Ospedaliero Universitaria Careggi -	42	24	57.1

Interventistica cardiologica strutturale			
Ospedale Civile Sant'Andrea	56	33	58.9
Azienda Ospedaliero Universitaria Careggi - Cardiologia interventistica d'urgenza	25	15	60.0
Azienda Ospedaliero Universitaria Policlinico Paolo Giaccone	12	8	66.7
Ospedale degli Infermi	12	8	66.7
Azienda Ospedaliera "G. Brotzu"	52	41	78.8
Ospedale di Conegliano	34	27	79.4
Ospedale "Di Venere"	15	12	80.0
Azienda Ospedale-Universita Padova	18	16	88.9
IRCCS Ospedale Galeazzi - Sant'Ambrogio	2	2	100.0
Centro Unico di Emodinamica Rivoli-San Luigi Orbassano	30	30	100.0

Center-level median imaging uptake was 49.2% (IQR 37.8% to 72.8%; range 16.7% to 100.0%).

Assessment of barriers limiting Optical Coherence Tomography penetration in Actual Clinical practice & Training (OCT2ACT) study

OBSERVATIONAL, PROSPECTIVE, MULTICENTER, INVESTIGATOR-DRIVEN STUDY

CONFIDENTIAL

Clinical Study Protocol

ID PROTOCOL: OCT2ACT

VERSION 1.0, January 31th 2025

Sponsor Consorzio Futuro in Ricerca (CFR)

Via Saragat, 1 - 44122 Ferrara (FE) – Italy

Study Chairman

Prof. Simone Biscaglia

U.O. di Cardiologia, Azienda Ospedaliero Universitaria di Ferrara - Via Aldo Moro, 8 – 44124 Cona (FE) – Italy

Study Co-Chair

Dr. Rocco Vergallo

Cardiothoracic and Vascular Department (DICATOV)
Ospedale Policlinico San Martino IRCCS, Largo R. Benzi, 10 – 16132 Genova (GE) – Italy

Dr. Paolo Canova

USD Cardiologia 3 Diagnostica Interventistica, Dipartimento Cardiovascolare,
ASST Papa Giovanni XXIII, P.zza OMS, 1 – 24127 Bergamo (BG) – Italy

Coordinating center

Principal Investigator: Dr. Giuseppe Vadalà

U.O.C. di Cardiologia
Azienda Ospedaliero Universitaria Policlinico Paolo Giaccone, Via del Vespro, 129 – 90127 Palermo (PA) – Italy

I. PROTOCOL APPROVAL PAGE

Sponsor:

Name: Prof. Donato Vincenzi — Role: President Consorzio Futuro in Ricerca (CFR)

Date: — Signature:

Study Chairman:

Name: Dr. Simone Biscaglia — Role: Study Chair

Date: — Signature:

II. SIGNATURE PAGE PRINCIPAL INVESTIGATOR

**Study title:**	Assessment of barriers limiting **O**ptical **C**oherence **T**omography penetration in **A**ctual **C**linical practice & **T**raining (OCT2ACT) study
**Id Protocol:**	OCT2ACT
**Version no.:**	1.0
**Date:**	January 31st, 2025
**Study type:**	Post-marketing observational study with medical device

I have read this protocol and/or amendment and appendices and agree to adhere to the requirements. I will provide copies of this protocol and all pertinent information to the study personnel under my supervision. I will discuss this material with them and ensure they are fully informed regarding the investigational product and the conduct of the study.

I agree to conduct this study according to this protocol and to comply with its requirements, subject to ethical and safety considerations and guidelines, and to conduct the study in accordance with ISO 14155 and applicable regional regulatory requirements.

Name: Role: Principal Investigator

Date: Signature:

III. PROTOCOL SYNOPSIS

**Study Title**	Assessment of barriers limiting Optical Coherence Tomography penetration in Actual Clinical practice & Training (OCT2ACT) study
**ID Protocol**	OCT2ACT
**Study type**	This is an observational, prospective, multicenter, investigator-driven study with post-market medical devices, not intended for CE marking.
**No. of sites involved**	27
**No. of patients**	1000
Introduction	
**Study Background**	The updated guidelines from the European Society of Cardiology (ESC) for chronic coronary syndrome (CCS) have upgraded the use of IVUS and OCT to a class IA recommendation for complex PCI, based on evidence showing a reduction in serious clinical events, including mortality, compared to conventional angiography alone. Despite this, IVUS and OCT are underutilized in daily practice due to factors such as time, cost, and limited technology availability. Investigating the reasons behind this underuse is necessary, especially now that these technologies are more accessible and cost-effective. Additionally, OCT could be particularly helpful in specific cases such as coronary bifurcations and severe calcifications, warranting further evaluation of its use in these settings.
**Study Objective(s)**	The objective of the study OCT2ACT is to investigate:<br>• The main reasons/barriers limiting the use of intracoronary imaging in complex PCI cases where it is indicated as suggested by guidelines;<br>• The main reasons behind the selection of IVUS or OCT in such patients;<br>• In cases where OCT is used, how this technology can

	influence the procedural planning and optimization in complex clinical settings.
**Study Endpoint(s)**	**Endpoints**<br>*1. Cohort intracoronary imaging NO*<br>• Primary endpoint:<br>- Reasons/barriers for not using intracoronary imaging.<br><br>As suggested in the consensus document by Cabana et al, the main reasons/barriers will be classified according to the following groups:<br><br>**Attitudes (lack of agreement and inertia of previous practice):**<br>- Clinical and angiographic data are sufficient<br>- Intracoronary imaging would not change my PCI strategy<br>- Intracoronary imaging has low added value on improving clinical outcome<br><br>**Knowledge (lack of awareness and familiarity):**<br>- Intracoronary imaging not feasible for technical or anatomical reasons (e.g., tortuous vessels, etc.)<br>- Lack of confidence in interpretation of images<br>- Lack of confidence in translating imaging features into an actionable PCI plan<br>- Inadequate support by device specialists in the cath lab<br><br>**Behavior (external barriers):**<br>- Time constraint<br>- Device costs<br>- Absence of adequate coding / reimbursement<br><br>• Secondary endpoints:<br>- Percentage of PCI matching inclusion/exclusion criteria;<br>- Percentage of PCI where intracoronary imaging is used as compared to overall volume of PCI;<br>- Percentage of PCI where intracoronary imaging is used as compared to overall volume of PCI matching inclusion/exclusion criteria;<br>- Association between baseline characteristics and missing use of intracoronary imaging;

	- Association between procedural characteristics and missing use of intracoronary imaging.<br><br>*2. Cohort intracoronary imaging YES according to guidelines recommendations*<br>• Primary endpoint:<br>- Reasons behind the selection of IVUS or OCT.<br><br>As suggested in the consensus document by Cabana et al, the main reasons/barriers will be classified according to the groups already mentioned for the previous cohort (Attitudes, Knowledge, Behavior).<br><br>*3. Cohort intracoronary imaging YES outside guidelines recommendations*<br>• Primary endpoint:<br>- Reasons behind the selection of OCT or IVUS outside guidelines recommendations.<br><br>• Safety endpoints:<br>- Procedural time;<br>- Contrast media amount;<br>- Procedural complications related to OCT use.<br>The safety endpoints will be recorded in both the cohorts intracoronary imaging YES and compared to investigate differences.<br><br>• Secondary endpoints:<br>- Association between baseline characteristics and IVUS preference;<br>- Association between procedural characteristics and IVUS preference;<br>- Association between baseline characteristics and OCT preference;<br>- Association between procedural characteristics and OCT preference;<br>- Description of timing and modality of the intracoronary imaging;<br>- Description of the operator's perceived advantages by intracoronary imaging use;<br>- Description of OCT penetration over IVUS in bifurcations,

	LM and stent failure.<br><br>The secondary endpoints will be recorded in both the cohorts intracoronary imaging YES and compared to investigate differences.
Methods	
**Study design**	OCT2ACT is an observational, prospective, multicenter, investigator-driven study with post-market medical devices.<br>The participating centers will include three cohorts of patients:<br><br>**1. Cohort intracoronary imaging NO:** patients who meet the inclusion/exclusion criteria, but did not undergo intracoronary imaging (IVUS or OCT) due to operator's decision.<br>The aim is to describe the main reasons/barriers for not using imaging.<br><br>**2. Cohort intracoronary imaging YES according to guidelines recommendation:** patients who meet the inclusion/exclusion criteria and underwent intracoronary imaging as recommended by guidelines.<br>The aim is to understand the main reasons for the decision to use IVUS or OCT and the clinical benefits perceived by the operator.<br><br>**3. Cohort intracoronary imaging YES outside guidelines recommendation:** patients who meet the inclusion/exclusion criteria and underwent intracoronary imaging (IVUS or OCT) outside guideline indications.<br>The aim is to understand the main reasons for the decision to use IVUS or OCT outside the guideline indications, its safety and the perceived clinical benefits for the operator.
**Study procedures**	The pseudonymised data will be collected during a 3-month window through an electronic Case Report Form. Operators will complete the eCRF at the end of each procedure that meets the inclusion/exclusion criteria, detailing the reasons for using or not using imaging.
**Study population**	*Inclusion and Exclusion Criteria*<br>Patients ≥ 18 years will be enrolled according to the different

<table>
<tr>
<td></td>
<td>
study cohorts:
<u>1. Cohort intracoronary imaging NO</u>
<u>Inclusion criteria</u>
Patients undergoing clinically indicated PCI and having at least one of the following criteria (suggestive for complex PCI) without receiving intracoronary imaging:
- Long lesion (>38 mm)
- True bifurcation involving side-branch with a reference diameter of >2.5 mm and Medina 1.1.1
- Left main bifurcation
<u>Exclusion criteria</u>
- Refusal of informed consent
<u>2. Cohort intracoronary imaging YES according to guidelines recommendation</u>
<u>Inclusion criteria</u>
Patients undergoing clinically indicated PCI with the guidance of intracoronary imaging (OCT or IVUS) and having at least one of the following criteria (suggestive for complex PCI) receiving imaging:
- Long lesion (>38 mm)
- True bifurcation involving side-branch with a reference diameter of >2.5 mm and Medina 1.1.1
- Left main bifurcation
<u>Exclusion criteria</u>
- Refusal of informed consent
<u>3. Cohort intracoronary imaging YES outside guidelines recommendation</u>
<u>Inclusion criteria</u>
Patients undergoing OCT/IVUS use.
<u>Exclusion criteria</u>
- Long lesion (>38 mm)
- True bifurcation involving side-branch with a reference diameter of >2.5 mm and Medina 1.1.1
- Left main bifurcation
- Refusal of informed consent
</td>
</tr>
<tr>
<td>Variables</td>
<td>- Reasons for using or not using imaging (see above)</td>
</tr>
</table>

	- Clinical benefit perceived by the operator<br>- Reasons for selecting one or other imaging modality
**Sample size**	A formal sample size calculation is not necessary for this study. We aim to recruit at least 20 hub centres performing 70 PCI per month, with each centre contributing at least 90 complex PCI cases, leading to a total of at least 1000 cases.
**Statistical analysis**	A Detailed Statistical Analysis Plan will be completed before recruitment ends. Normality of continuous variables will be assessed, with comparisons using Student's t-test or Wilcoxon's test. Categorical variables will be analysed using Pearson's chi-square or Fisher's exact test. Multivariable logistic models will identify variables associated with the use of intracoronary imaging. Statistical analysis will be performed using STATA version 16, with a significance level of $p < 0.05$.

IV. TABLE OF CONTENTS

I. PROTOCOL APPROVAL PAGE 3

II. SIGNATURE PAGE PRINCIPAL INVESTIGATOR 4

III. PROTOCOL SYNOPSIS 5

IV. TABLE OF CONTENTS 11

Study design 13

Setting 14

Inclusion/exclusion criteria 15

Endpoints 17

Data collection 20

Statistical analysis 20

Sample size 20

Good Clinical Practice 20

Informed Consent of the Patient 21

Approval of the Study Protocol 21

Maintenance of Records 21

Confidentiality 22

Curriculum vitae 22

Confidentiality agreement 22

Ownership of data 22

Publications 23

Bias 23

Insurance 23

References 24

Background

The last update of European Society of Cardiology (ESC) chronic coronary syndrome (CCS) guidelines upgraded the use of intravascular ultrasound (IVUS) imaging and optical coherence tomography (OCT) to class IA in the setting of complex percutaneous coronary intervention (PCI) (in particular left main, true bifurcations, and long lesions).

Assessment of procedural risks and post-procedural outcomes		
Intracoronary imaging guidance by IVUS or OCT is recommended for performing PCI on anatomically complex lesions, in particular left main stem, true bifurcations and long lesions.	I	A

This strong level of recommendation is based on the recent evidence that intracoronary imaging reduces the incidence of hard clinical endpoints, including mortality, as compared to conventional angiography alone.

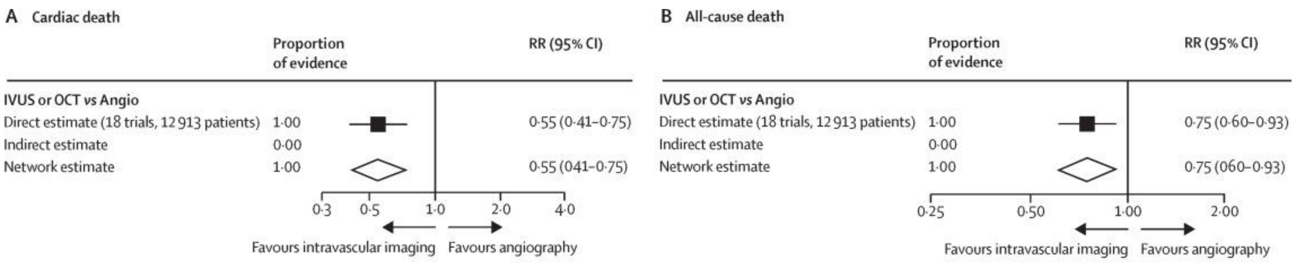

Yet, there is compelling evidence documenting the underutilization of IVUS and OCT in daily practice. Many potential reasons, including time, costs, limited availability of technology, expertise of the operators have been suggested. However, no specific study investigated the reasons behind this malpractice. In particular, these reasons should be investigated now that intracoronary imaging has been upgraded to class I recommendation and that many technologies are available significantly reducing the economic burden. In addition, there are some particular anatomic subsets where OCT could be particularly helpful (bifurcations, severe calcifications, acute coronary syndrome patients) and it could be interesting to assess the penetration of this technology in this subset of patients. Finally, these devices can be used also in anatomical subsets (diagnosis and procedural guidance) outside from Guidelines recommendations and it is important to assess their safety and clinical advantage in this setting.

Objective

The OCT2ACT study aims to investigate:

- The main reasons/barriers limiting the use of intracoronary imaging in complex PCI where it is indicated as suggested by guidelines
- The main reasons behind the selection of IVUS or OCT in complex PCI
- In complex PCI guided by OCT, How OCT can influence the procedural planning and optimization in complex clinical settings.

Study design

The OCT2ACT is an observational, prospective, multicenter, investigator-driven study. Participating centers will include three different cohorts of patients:

1. *Cohort intracoronary imaging NO*

In this cohort, the investigators will include all the patients matching inclusion/exclusion criteria, but in whom intracoronary imaging (IVUS or OCT) was not used (operator's decision). The aim is to have a description of the main reasons/barriers that led to the avoidance of imaging utilization.

2. *Cohort intracoronary imaging YES according to guidelines recommendation*

In this cohort, the investigators will include all the patients matching inclusion/exclusion criteria in whom intracoronary imaging (IVUS or OCT) was used (operator's decision) according to guidelines recommendation. The aim is to understand the main reasons behind the decision to use IVUS or OCT and the clinical advantages perceived by the operators.

3. *Cohort intracoronary imaging YES outside guidelines recommendation*

In this cohort, the investigators will include all the patients matching inclusion/exclusion criteria in whom OCT was used (operator's decision) outside Guidelines recommendations. The aim is to understand the main reasons behind the decision to use OCT outside guidelines recommendation, its safety and the clinical advantages perceived by the operators.

Setting

After Ethic Committee approval and site initiation visit, each participating center will recruit consecutive patients for a time window of 3 months.

Inclusion/exclusion criteria

Patients ≥ 18 years will be enrolled according to the different study cohorts:

1. *Cohort intracoronary imaging NO*
    - *Inclusion criteria*

Patients undergoing clinically indicated PCI and having at least one of the following criteria (suggestive for complex PCI) without receiving intracoronary imaging:

- Long lesion (>38 mm)
- True bifurcation involving side-branch with a reference diameter of >2.5 mm and Medina 1.1.1
- Left main bifurcation

- *Exclusion criteria*

- Refusal of informed consent

2. *Cohort intracoronary imaging YES according to guidelines recommendation*
    - *Inclusion criteria*

Patients undergoing clinically indicated PCI with the guidance of intracoronary imaging (OCT or IVUS) and having at least one of the following criteria (suggestive for complex PCI) receiving imaging:

- Long lesion (>38 mm)
- True bifurcation involving side-branch with a reference diameter of >2.5 mm and Medina 1.1.1
- Left main bifurcation

- *Exclusion criteria*

- Refusal of informed consent

3. *Cohort intracoronary imaging YES outside guidelines recommendation*
    - *Inclusion criteria*

Patients undergoing OCT use.

- *Exclusion criteria*

- Long lesion (>38 mm)
- True bifurcation involving side-branch with a reference diameter of >2.5 mm and Medina 1.1.1
- Left main bifurcation
- Refusal of informed consent

Endpoints

4. Cohort intracoronary imaging NO

- Primary endpoint:

- Reasons/barriers for not using intracoronary imaging.

As suggested in the consensus document by Cabana et al, the main reasons/barriers will be classified according to the following groups:

Attitudes (lack of agreement and inertia of previous practice):

- Clinical and angiographic data are sufficient
- Intracoronary imaging would not change my PCI strategy
- Intracoronary imaging has low added value on improving clinical outcome

Knowledge (lack of awareness and familiarity):

- Intracoronary imaging not feasible for technical or anatomical reasons (e.g., tortuous vessels, etc.)
- Lack of confidence in interpretation of images
- Lack of confidence in translating imaging features into an actionable PCI plan
- Inadequate support by device specialists in the cath lab

Behavior (external barriers):

- Time constraint
- Device costs
- Absence of adequate coding / reimbursement

- Secondary endpoints:

- Percentage of PCI matching inclusion/exclusion criteria;
- Percentage of PCI where intracoronary imaging is used as compared to overall volume of PCI;
- Percentage of PCI where intracoronary imaging is used as compared to overall volume of PCI matching inclusion/exclusion criteria;

- Association between baseline characteristics and missing use of intracoronary imaging;
- Association between procedural characteristics and missing use of intracoronary imaging.

5. Cohort intracoronary imaging YES according to guidelines recommendations

- Primary endpoint:
- Reasons behind the selection of IVUS or OCT.

As suggested in the consensus document by Cabana et al, the main reasons/barriers will be classified according to the groups already mentioned for the previous cohort (Attitudes, Knowledge, Behavior).

6. Cohort intracoronary imaging YES outside guidelines recommendations

- Primary endpoint:
- Reasons behind the selection of OCT or IVUS outside guidelines recommendations.

- Safety endpoints:
- Procedural time;
- Contrast media amount;
- Procedural complications related to OCT or IVUS use.

The safety endpoints will be recorded in both the cohorts intracoronary imaging YES and compared to investigate differences.

- Secondary endpoints:
- Association between baseline characteristics and IVUS preference;
- Association between procedural characteristics and IVUS preference;
- Association between baseline characteristics and OCT preference;
- Association between procedural characteristics and OCT preference;
- Description of timing and modality of the intracoronary imaging;
- Description of the operator's perceived advantages by intracoronary imaging use;
- Description of OCT penetration over IVUS in bifurcations, LM and stent failure.

The secondary endpoints will be recorded in both the cohorts intracoronary imaging YES and compared to investigate differences.

Data collection

All study data will be pseudo anonymised and collected in a web-based electronical case report form. An academic research organization will monitor integrity and quality of the data.

Statistical analysis

A detailed Statistical Analysis Plan will be finalized before the end of the recruitment. Briefly, the normal distribution of continuous variables will be assessed through the Shapiro-Wilk test. Continuous variables will be summarized as means (SD) or median [IQR], and comparisons will be made using the Student t-test or the Wilcoxon test, as appropriate. Categorical variables will be presented as frequencies and percentages, and comparative analyses will be conducted using either the Pearson Chi-square or Fisher's exact test, as deemed appropriate. Multivariable logistic models will be built to discriminate the variables associated with use or not of intracoronary imaging. Statistical analyses will be performed using the STATA statistical software version 16 (StataCorp LLC, College Station, TX, USA). We will perform two-tailed analysis and we will consider a p value less than 0.05 to be significant.

Sample size

Based on the nature of the study, a formal sample size computation is not warranted. Anyway, we suppose to recruit at least 20 hub centers performing at least 70 PCI per month. Based on inclusion/exclusion criteria, we suppose that each center will perform at least 30 complex PCI by month. Therefore, any center will contribute with at least 90 cases, configuring an overall population of at least 1000 cases.

Good Clinical Practice

The procedures set out in this protocol are designed to ensure that the investigator abides by the principles of the Declaration of Helsinki and Good Clinical Practice Guidelines (ICH-GCP) in the latest version, in the conduct, evaluation, and documentation of the study. A

copy of these documents will be provided to each center. The study will be carried out according to local legal requirements and international regulations.

Informed Consent of the Patient

Patients who meet all inclusion criteria and none of the exclusion criteria are deemed eligible. Before being enrolled into the clinical study, the patient must provide written consent to participate in the study after the nature, scope and possible consequences of the clinical study have been explained both orally and in writing. All patients who signed informed consent must be listed on the Screening Log.

Approval of the Study Protocol

The present study is configured as a post-marketing observational study with medical device. For this reason, the Ministry of Health Circular of 28/02/2023 can be applied, and the approval of the Coordinating Ethical Committee will be obtained before the start of the study. To this end, the study protocol and the informed consent form used at the site and other appropriate documents must be submitted and approved by the Coordinating Ethics Committee and notified to the Ministry of Health according to local requirements. The approval from that EC should be applied to all the participating centers, according to the law.

Maintenance of Records

The Investigator agrees to obtain a correctly completed informed consent form for each patient included in the study. The investigator will maintain a personal list of patient numbers and patient names to allow records to be found later. The Investigator must maintain all study records, patient files and other source data for the maximum period permitted by the hospital, institution, or private practice. However national regulations should be considered, and the longest time allowed by these rules would be counted. For trials conducted in the European Community, the Investigator is required to arrange for the retention of patient identification codes for at least 7 years after the completion or discontinuation of the trial.

Confidentiality

Patients will be identified throughout documentation and evaluation by the number assigned to them by the study. Patients will be assured that all findings will be stored on the computer and handled with the strictest confidence. The Investigator agrees to maintain the confidentiality of the study protocol.

Curriculum vitae

An updated copy of the curriculum vitae for each Investigator and co-Investigator will be provided prior to the beginning of the study.

Confidentiality agreement

All goods, materials, information (oral or written) and unpublished documentation provided to the Investigators (or any company acting on their behalf), inclusive of this protocol and the patient case report forms are the exclusive property of the Cardiovascular Department of the University of Ferrara. They may not be given or disclosed by the Investigator or by any person within his authority, either in part or in totality, to any unauthorized person without the prior written formal consent. It is specified that the submission of this protocol and other necessary documentation to the EC or a-like body (IRB, CCPPRB...) is expressly permitted, the Ethics Committee members having the same obligation of confidentiality. The Investigator shall consider as confidential and shall take all necessary measures to ensure that there is no breach of confidentiality in respect of all information accumulated, acquired or deduced in the course of the trial, other than that information to be disclosed by law.

Ownership of data

The Sponsor has ownership of all data and results collected during this study. The full publication rights of the study data reside solely with the Principal Investigator.

Publications

All study presentations and/or publication of the results will be based on clean, checked and validated data in order to ensure the accuracy of the results. Publication of the main findings of this study will be made based on the contributions of individuals to the overall study. All the trial participants (investigators and committee members) make a prior delegation of responsibility for primary presentation and/or primary publication of the results to the Steering Committee.

Bias

The present study is observational and it is aimed to picture the clinical routine in a prespecified time span. Therefore, no specific bias are identifiable.

Insurance

The study is an observational study. The protocol does not change clinical practice and patient's management. The protocol does not influence the application of tools, devices and drugs. For these reasons, no specific insurance is needed.

References

1. Vrints C, Andreotti F, Koskinas KC, Rossello X, Adamo M, Ainslie J, Banning AP, Budaj A, Buechel RR, Chiariello GA, Chieffo A, Christodorescu RM, Deaton C, Doenst T, Jones HW, Kunadian V, Mehilli J, Milojevic M, Piek JJ, Pugliese F, Rubboli A, Semb AG, Senior R, Ten Berg JM, Van Belle E, Van Craenenbroeck EM, Vidal-Perez R, Winther S; ESC Scientific Document Group. 2024 ESC Guidelines for the management of chronic coronary syndromes. Eur Heart J. 2024 Sep 29;45(36):3415-3537. doi: 10.1093/eurheartj/ehae177. PMID: 39210710.

2. Stone GW, Christiansen EH, Ali ZA, Andreasen LN, Maehara A, Ahmad Y, Landmesser U, Holm NR. Intravascular imaging-guided coronary drug-eluting stent implantation: an updated network meta-analysis. Lancet. 2024 Mar 2;403(10429):824-837. doi: 10.1016/S0140-6736(23)02454-6. Epub 2024 Feb 21. PMID: 38401549.
